# Neuronal Circuits Supporting Development of Visual Naming Revealed by Intracranial Coherence Modulations

**DOI:** 10.3389/fnins.2022.867021

**Published:** 2022-05-19

**Authors:** Ravindra Arya, Brian Ervin, Jason Buroker, Hansel M. Greiner, Anna W. Byars, Leonid Rozhkov, Jesse Skoch, Paul S. Horn, Clayton Frink, Craig Scholle, James L. Leach, Francesco T. Mangano, Tracy A. Glauser, Katherine D. Holland

**Affiliations:** ^1^Division of Neurology, Comprehensive Epilepsy Center, Cincinnati Children’s Hospital Medical Center, Cincinnati, OH, United States; ^2^Department of Pediatrics, University of Cincinnati College of Medicine, Cincinnati, OH, United States; ^3^Department of Electrical Engineering and Computer Science, University of Cincinnati, Cincinnati, OH, United States; ^4^Division of Pediatric Neurosurgery, Cincinnati Children’s Hospital Medical Center, Cincinnati, OH, United States; ^5^Division of Pediatric Neuroradiology, Cincinnati Children’s Hospital Medical Center, Cincinnati, OH, United States

**Keywords:** language development, brain networks, intracranial EEG, neuronal connectivity, stereo EEG

## Abstract

**Background:**

Improvement in visual naming abilities throughout the childhood and adolescence supports development of higher-order linguistic skills. We investigated neuronal circuits underlying improvement in the speed of visual naming with age, and age-related dynamics of these circuits.

**Methods:**

Response times were electronically measured during an overt visual naming task in epilepsy patients undergoing stereo-EEG monitoring. Coherence modulations among pairs of neuroanatomic parcels were computed and analyzed for relationship with response time and age.

**Results:**

During the overt visual naming task, mean response time (latency) significantly decreased from 4 to 23 years of age. Coherence modulations during visual naming showed that increased connectivity between certain brain regions, particularly that between left fusiform gyrus/left parahippocampal gyrus and left frontal operculum, is associated with improvement in naming speed. Also, decreased connectivity in other brain regions, particularly between left angular and supramarginal gyri, is associated with decreased mean response time. Further, coherence modulations between left frontal operculum and both left fusiform and left posterior cingulate gyri significantly increase, while that between left angular and supramarginal gyri significantly decrease, with age.

**Conclusion:**

Naming speed continues to improve from pre-school years into young adulthood. This age-related improvement in efficiency of naming environmental objects occurs likely because of strengthened direct connectivity between semantic and phonological nodes, and elimination of intermediate higher-order cognitive steps.

## Introduction

The ability to name objects in the visual environment (visual naming) is a fundamental skill that supports language development and the interface between language and cognition (Novack and Waxman, 2020). Naming ability expands rapidly in preschool years, with the vocabulary increasing from 50 to 100 words to over 2000 words from 2 to 5 years age (Dixon and Stein, 2006). Efficiency of word retrieval, which underlies naming ability, continues to further improve throughout the childhood and adolescence (Denckla and Rudel, 1974; Fried-Oken, 1984; Nippold, 2016). Efficient naming also supports acquisition of higher-order linguistic abilities such as syntax, grammar, and abstract representations (Nippold, 2016; Novack and Waxman, 2020). The importance of naming is underscored by the observation that dysnomia is a common feature of several disorders involving the cerebral cortex (Feldman, 2019; Ullman et al., 2020). Recognition of the fundamental role of visual naming has prompted research into determinants of naming accuracy and speed, its developmental trajectory, and impact of neurodevelopmental disorders (Nippold, 2016). Also, the functional neuroanatomy of visual naming has been defined using multiple modalities including lesion data, functional magnetic resonance imaging (fMRI), electrical stimulation mapping (ESM), and high-gamma modulation (HGM) topography (Naidich et al., 2001; Hamberger, 2007; Price, 2010; Arya et al., 2017).

However, the neuronal circuits that support development of efficient naming, and their age-related dynamics, remain poorly understood. Here, we tested the hypothesis that the response time (latency) during a visual naming task, an indicator of naming efficiency, will decrease with age. We further studied the association of response time with coherence modulations during visual naming between different cortical regions, to delineate the neuronal circuits supporting naming efficiency. Finally, we studied the relationship of age with coherence modulations for pairs of cortical regions having significant association with response time, to gain insight into age-related dynamics of these neuronal circuits. We hypothesized that increased synchronization of brain areas involved in sensory and motor aspects of visual naming with possible elimination of intermediate higher-order cognitive steps, may underlie age-related improvement in naming efficiency. We performed this work in patients with drug-resistant epilepsy (DRE) undergoing stereotactic electroencephalography (SEEG) monitoring harnessing its high spatiotemporal resolution, and the ability to use an overt naming task that allows accurate measurement of response latency.

## Materials and Methods

### Patients and Stereotactic Electroencephalography Acquisition

Patients with DRE undergoing SEEG monitoring at Cincinnati Children’s Hospital, who were native English speakers and able to participate in visual naming, were included. We excluded patients with (i) verbal comprehension index (VCI) < 70 measured using age-appropriate version of the Wechsler scales (Wechsler Preschool and Primary Scale of Intelligence, Wechsler Intelligence Scale for Children, or the Wechsler Adult Intelligence Scale), (ii) extensive lesions distorting the neuroanatomic landmarks, (iii) atypical (right/bilateral) language dominance, and (iii) age < 3 years (skull thickness often insufficient for SEEG implantation). Cerebral language dominance was obtained from fMRI if available, or left hemisphere was regarded as language dominant unless the patient was left-handed and had a left perisylvian developmental pathology (Nakai et al., 2017).

Stereotactic electroencephalography (SEEG) was monitored with electrodes having 0.86 mm diameter and 2.41 mm contacts, and sampled at 2048 Hz with Natus Quantum amplifier using Neuroworks 8.5 software (Natus Neuro, Middleton WI). For study purposes, SEEG was recorded with a referential montage, with the electrode contact farthest from the presumptive seizure-onset zone chosen as the reference. SEEG was recorded after the patients have recovered from the anesthesia and were off their regular anti-seizure medications. The study was approved by the institutional review board. Informed consent from patients ≥ 18 years-of-age and parental permission in others were obtained.

### Visual Naming Task and Response Times

A series of 40 colored diagrams was displayed on a monitor using E-Prime 3.0 software (Psychology Software Tools, Sharpsburg PA) (Arya et al., 2019b; Ervin et al., 2020). Each picture was shown for 2s (3s for patients < 8-years-of-age) with 1s interval. Patients were requested to name the picture aloud, immediately after the display. A trial run was performed, and the pictures that the patient was unable to name, were eliminated, to avoid unsuccessful trials. Then, the order of pictures was randomized before recording. Patient’s verbal output was collected with a microphone and routed through a digital trigger box to record the beginning and termination of patient’s voice on a channel synchronized to the SEEG data. Response onsets with –2 ≥ z-score ≥ 2 were excluded, because in our experience, these consist of filler words (e.g., “umm”) (Ervin et al., 2020). Mean response time, defined as the mean time between image display and onset of verbal response, was calculated for each patient.

### Stereotactic Electroencephalography Pre-processing

Stereotactic electroencephalography (SEEG) channels were rejected if their variance or point-to-point difference was an outlier from other channels in a given patient, as published by us previously (Ervin et al., 2020). Only electrode contacts localized to gray matter were analyzed, because the Medical Image Computing and Computer Assisted Intervention (MICCAI) atlas used by us, does not offer lobar/sub-lobar white matter parcelation. These channels were zero-phase notch filtered at harmonics of 60 Hz, and zero-phase band-pass filtered between 10 and 200 Hz, both using Hamming windows. Epochs were aligned starting 1 s prior to and continuing 2 s after the image display. These epochs were resampled at 683 Hz (3x down-sampling from the 2048 Hz sampling rate).

### Coherence Modulations

**FIGURE 1:**
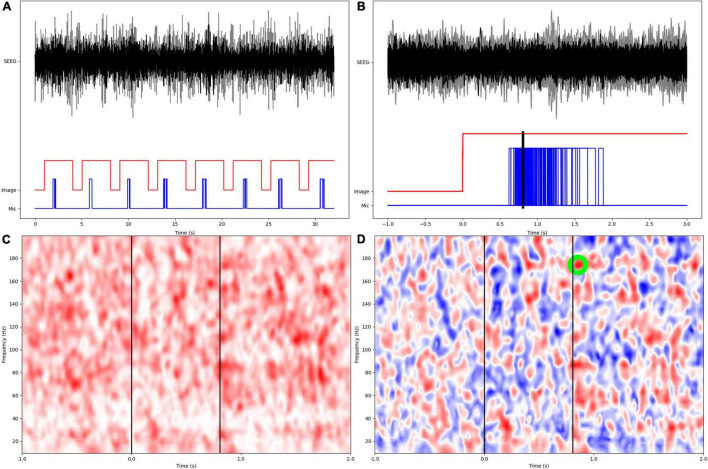
Overview of experimental design and signal processing. **(A)** SEEG data from two different anatomic parcels (black, gray), image display, and patient microphone digital input channels. **(B)** Epochs are aligned at the onset of image display, with a preceding 1s inter-trial baseline. Mean response time is calculated as arithmetic mean of times from image display to onset of verbal responses. **(C)** Wavelet based coherence calculation in the time-frequency space. **(D)** Coherence modulations are computed as z-scores based on distribution of coherence during the inter-trial baseline. The largest z-score within the naming phase (0–2s from image display) is identified and retained for subsequent statistical analyses.

We calculated broadband coherence modulations between the inter-trial baseline and the naming trial, as measures of synchrony between parcel pairs (Bastos and Schoffelen, 2015). Coherence is the frequency domain equivalent of cross-correlation in the time domain, and represents the amount of variance in one of a pair of signals, that can be explained by another signal, as a function of frequency. A time-frequency representation (TFR) of coherency was calculated for each pair of channels in 1 Hz bins using Morlet wavelet decomposition at *f*/5 cycles for each frequency *f*. The epochs were aligned with *t* = 0 at picture display, including 1s inter-trial baseline from the termination of the previous trial, excluding the first trial. The real valued coherence TFR was calculated as the magnitude of coherency (which is complex valued). Broadband coherence modulations were found by normalizing the inter-trial and naming trial phases by the mean and standard deviation (SD) of the coherence TFR during the inter-trial phase, relative to each frequency bin, converting coherence modulations into z-scores to ensure comparability across patients. Because of high likelihood of significant correlation among channels within the same anatomic parcel, we excluded such channel pairs. From the mean coherence modulation TFR of each parcel pair in every patient, we found the value of the maximum z-score during the averaged naming trial, with respect to the inter-trial baseline. Here, the naming trial was defined as 0–2s from the picture display including the times for verbal responses, thereby yielding a 0–2s x 10–200 Hz matrix for calculation of the maximum z-score. Also, the specific timings of the maximum z-scores for coherence modulations were not retained, only that they were computed during the naming trial (Figure 1). Coherence modulations were then averaged across all channels constituting a pair of neuroanatomic parcels.

### Image Processing

Pre-operative T1W-MRI and post-operative computed tomographic (CT) scan were co-registered to the pre-operative FLAIR using 6-parameter rigid body transformation. MRI images were non-linearly warped to the Montreal Neurological Institute (MNI) space using multi-channel segmentation. The same warping was then applied to the CT scan already co-registered to the MRI, with SPM12 toolbox in MATLAB. The electrode contacts were identified and labeled from the normalized CT scan, and assigned to a parcel within the MICCAI atlas, using the FASCILE software (Ervin et al., 2021b).

### Statistical Analysis

Because the electrode implantation is unique to each patient, we excluded pairs of neuroanatomic parcels contributed by < 5 patients, resulting in 365 parcel pairs. We first analyzed the relationship between mean response time and age using linear regression. Subsequently, linear regression models were fitted with mean response time as the dependent variable, and coherence modulations between each parcel pair as the explanatory variable. P-values for these linear models were penalized with family wise error (Šidák) correction because of high dimensionality of the feature space for coherence modulations between parcel pairs. To test the validity of our analysis, we created 5 simulated datasets by omitting 20% of randomly selected data (electrode contact level) each time and using multiple imputation with chained equations. We only retained parcel pairs with penalized p-values ≤ 0.05 for the relationship between coherence modulation and mean response time. Only for these parcel pairs, the relationship between coherence modulations and age was further analyzed using linear regression models.

## Results

**FIGURE 2:**
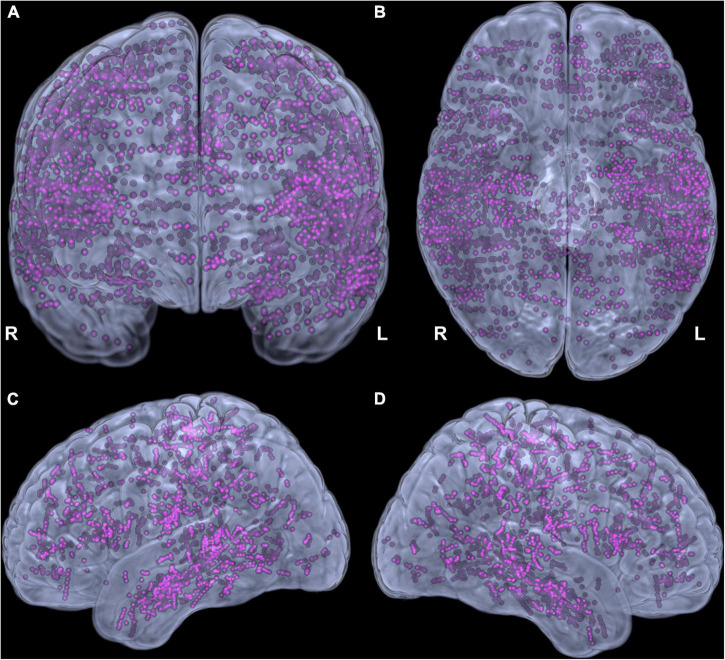
Adequacy of cortical sampling with stereotactic electrodes. Electrode contacts from all patients in the Montreal Neurological Institute space shown in coronal **(A)**, inferior **(B)**, left **(C)**, and right **(D)** views.

Forty patients (14 females) aged 3.9 to 23.1 years (mean ± standard deviation 13.5 ± 4.7) were studied. Four to 15 SEEG electrodes (11 ± 2) were implanted per patient, having 42 to 154 contacts (120 ± 26), with a total of 3590 contacts across all patients (Figure 2). Left, right, and both hemispheres were implanted in, respectively, 17, 13, and 10 patients. Language lateralization from fMRI was available in 28 patients (lateralization index 0.85 ± 0.19). VCI varied from 70 to 119 (90 ± 14). Brain MRI findings are summarized in Table 1. Additional clinical and demographic data are provided in Supplementary Table 1.

**TABLE 1:** Brain magnetic resonance imaging findings in all patients.

Brain MRI findings	Number
**Normal**	11
**Findings of uncertain significance** Incidental right frontal DVA (1) Non-specific signal in left ITG (1) Non-specific signal in left frontal/temporal white matter (1) Non-specific signal in right temporal lobe (1) T2/FLAIR hyperintensity in peri-ventricular white matter (3)	7
**Cortical dysplasia** Left hippocampus (2) Left parietal (1) Left temporal (1) Right parietal (1)	5
**Malformations of cortical development** Right hemisphere PMG (1) Right hemisphere PMG and heterotopia (1)	2
**Mesial temporal sclerosis** Left (3) Right (1)	4
**Gliosis ± encephalomalacia** Bilateral occipital (1) Bilateral frontal (1) Bilateral temporal (1) Bilateral peri-ventricular (1) Left peri-atrial (2) Right insula (1) Right peri-ventricular (2)	9
**Vascular** Multiple cavernoma (1)	1
**Neoplastic** Glioma involving optic chiasma and hypothalamus (1)	1
**Total**	40

*DVA, Developmental Venous Anomaly; FLAIR, Fluid Attenuation Inversion Recovery; ITG, Inferior Temporal Gyrus; MRI, Magnetic Resonance Imaging; PMG, Polymicrogyria.*

### Mean Response Time and Age

**FIGURE 3:**
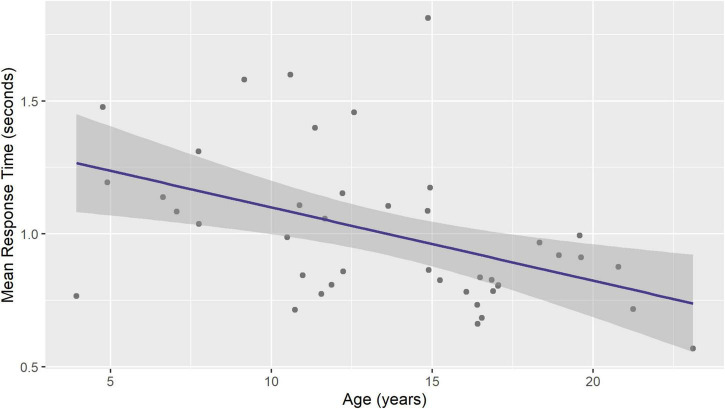
Mean response time during visual naming decreases with age. Figure shows ordinary least squares linear regression line with 95% standard error band.

The mean response time varied from 0.6 to 1.8 s (1.0 ± 0.3). Significant age-related decrease was seen in mean response time (regression coefficient –0.03, 95% CI –0.04, –0.01, *p* = 0.0024, Figure 3).

### Mean Response Time and Coherence Modulations

**FIGURE 4:**
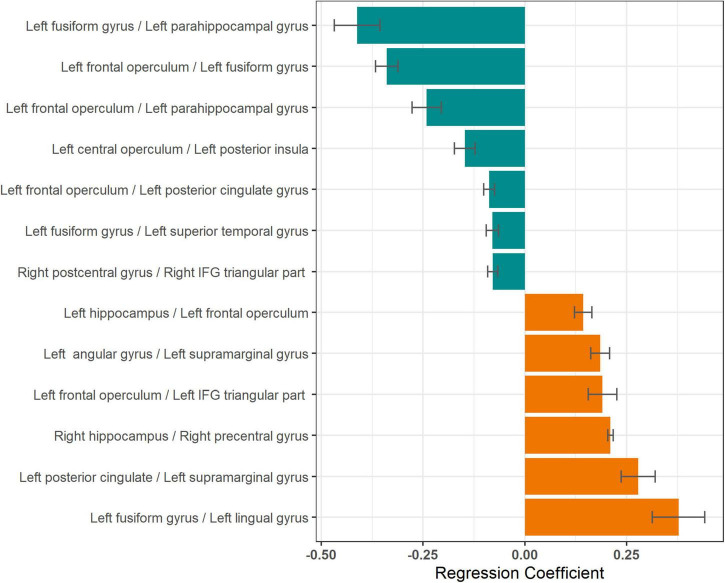
Relationship between mean response times and coherence modulations between pairs of neuroanatomic parcels. Lengths of the bars represent the regression coefficients (slopes) for linear models with mean response time as the dependent variable and coherence modulations between pairs of neuroanatomic parcels as the independent variable. Negative regression coefficients (green bars) imply decrease in response time (improving naming speed) with increase in coherence modulation for respective pairs. Only parcel pairs with *p* ≤ 0.05, after correction for multiple comparisons, are shown.

In individual patients, 31 ± 6 naming trials were averaged for computing coherence modulations. Coherence modulations in 13 parcel pairs showed a significant relationship with mean response time, after adjusting for multiple models (Table 2). An increase in coherence modulations between left fusiform and left parahippocampal gyri; left frontal operculum and left fusiform gyrus; left frontal operculum and left parahippocampal gyrus; left central operculum and left posterior insula; left frontal operculum and left posterior cingulate gyrus; left fusiform and left superior temporal gyri; and right postcentral and pars triangularis of right inferior frontal gyri (IFG), were associated with a decrease in mean response time. However, decrease in coherence modulations between left hippocampus and left frontal operculum; left angular and left supramarginal gyri; left frontal operculum and left IFG pars triangularis (MICCAI parcelation regards IFG pars triangularis as distinct from frontal operculum), left posterior cingulate and left supramarginal gyrus; left fusiform and left lingual gyri; and right hippocampus and right precentral gyrus, were associated with a decrease in mean response time (Figure 4). Coherence modulations in these 13 parcel pairs were consistently found to have significant relationship with mean response time in all simulated datasets.

**TABLE 2:** Pairs of brain parcels showing significant relationship between mean response time (response latency) and coherence modulations, and relationship between coherence modulations and age in those parcels.

Parcel pair	Mean response time ∼ coherence modulation		Coherence modulation ∼ age	
	Regression coefficient (95% CI)	p-value*	Regression coefficient (95% CI)	p-value
Left fusiform gyrus/Left parahippocampal gyrus	–0.41 (–0.72, –0.10)	0.0210	0.016 (–0.17, 0.21)	0.8226
Left frontal operculum/Left fusiform gyrus	–0.34 (–0.51, –0.16)	0.0085	0.10 (0.01, 0.19)	**0.0130**
Left frontal operculum/Left parahippocampal gyrus	–0.24 (–0.44, –0.04)	0.0289	0.12 (–0.07, 0.32)	0.1541
Left central operculum/Left posterior insula	–0.15 (–0.26, –0.04)	0.0147	0.05 (–0.13, 0.24)	0.5538
Left frontal operculum/Left posterior cingulate gyrus	–0.09 (–0.16, –0.02)	0.0209	0.30 (0.08, 0.52)	**0.0178**
Left fusiform gyrus/Left superior temporal gyrus	–0.08 (–0.15, –0.01)	0.0289	0.04 (–0.33, 0.42)	0.8042
Right postcentral gyrus/Right IFG triangular part	–0.08 (–0.14, –0.02)	0.0192	0.05 (–0.42, 0.51)	0.8170
Left hippocampus/Left frontal operculum	0.14 (0.04, 0.25)	0.0191	0.02 (–0.51, 0.54)	0.9420
Left angular gyrus/Left supramarginal gyrus	0.19 (0.06, 0.31)	0.0151	–0.22 (–0.41, –0.02)	**0.0096**
Left frontal operculum/Left IFG triangular part	0.19 (0.03, 0.35)	0.0233	0.06 (–0.17, 0.30)	0.5526
Right hippocampus/Right precentral gyrus	0.21 (0.17, 0.25)	0.0001	–0.09 (–0.40 0.21)	0.4482
Left posterior cingulate/Left supramarginal gyrus	0.28 (0.05, 0.51)	0.0290	–0.13 (–0.36, 0.10)	0.1846
Left fusiform gyrus/Left lingual gyrus	0.38 (0.07, 0.68)	0.0226	–0.04 (–0.15, 0.07)	0.4247

*p-values for mean response time as a function of coherence modulations were penalized for multiple comparisons, while those for coherence modulations as a function of age were not, because the latter analysis was limited to only these 13 parcel-pairs.

*CI, Confidence Interval; IFG, Inferior Frontal Gyrus.*

**FIGURE 5:**
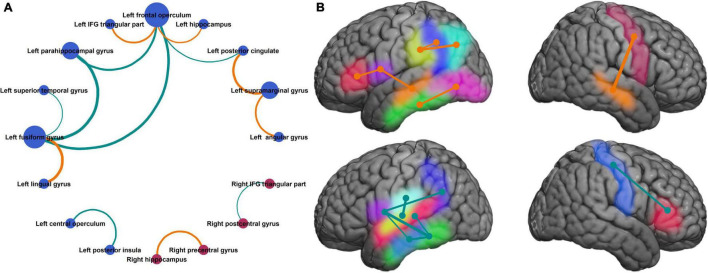
Neuronal circuits supporting improvement in naming speed. **(A)** Neuroanatomic parcel pairs where coherence modulation was significantly related to mean response time, show one predominant network with a closed loop between left fusiform gyrus, left parahippocampal gyrus, and left frontal operculum. **(B)** Same parcels shown in the Montreal Neurological Institute brain space. Parcels on medial surfaces of the cerebral hemispheres are projected to respective dorsolateral surfaces. [**(A)** Bubble size represents degree of the node, bubble color represents the hemisphere (blue = left, red = right), edge width represents absolute value of slope for the linear regression (same as Figure 4), and edge color represents the sign of regression coefficient (same as in Figure 4). **(B)** Left hemisphere (top panel): inferior frontal gyrus pars triangularis (red), frontal operculum (purple), supramarginal gyrus (yellow), angular gyrus (cyan), posterior cingulate (blue), hippocampus (orange), fusiform gyrus (green), lingual gyrus (pink). Left hemisphere (bottom panel): central operculum (sky blue), posterior insula (lime), superior temporal gyrus (deep pink), parahippocampal gyrus (light blue), rest same as top panel. Right hemisphere: hippocampus (yellow), precentral gyrus (red), postcentral gyrus (blue), inferior frontal gyrus pars triangularis (green). Edge colors and thickness same as Figures 3, 4].

On plotting a network graph of the parcels with significant association between coherence modulations and mean response time, four subnetworks were seen (Figure 5). It is evident from Figure 5, that there is one predominant subnetwork containing 10 notes and having left fusiform gyrus and left frontal operculum as hubs (significantly higher degree than other nodes) which form a closed loop with left parahippocampal gyrus. The other 3 subnetworks each have only a single edge connecting 2 nodes.

### Coherence Modulations and Age

**FIGURE 6:**
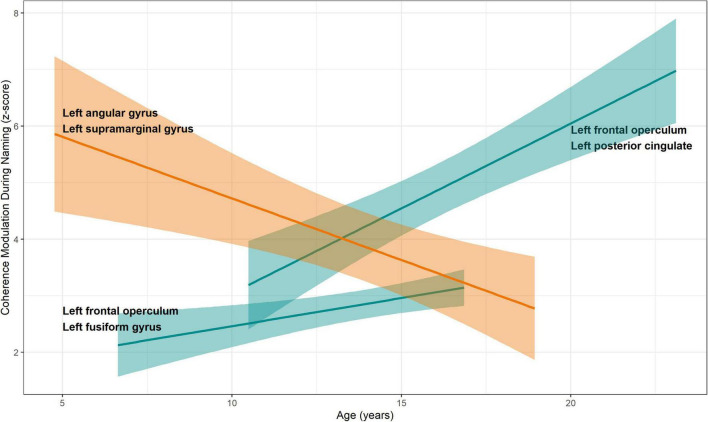
Relationship between coherence modulations in pairs of neuroanatomic parcels and age. Linear regression models were fitted for coherence modulation as a function of age, for parcel pairs where coherence modulations were significantly related to mean response times. A significant increase was seen for pairs of left frontal operculum with left fusiform gyrus and left posterior cingulate, respectively. Increased coherence modulations in these pairs were associated with reduced mean response time (better naming speed, see Figure 4). Coherence modulations between left angular and left supramarginal gyri decreased with age. Also, decreased coherence modulation between these parcels was associated with decrease in mean response time. Color scheme same as Figure 4. 95% standard error bands shown around regression lines.

Among the aforementioned 13 parcel pairs, 3 showed a significant age-related change (Figure 6). Coherence modulations between left frontal operculum and left fusiform gyrus, and between left frontal operculum and left posterior cingulate, significantly increased with age, whereas that between left angular and supramarginal gyri decreased with age (Table 2).

## Discussion

We found that naming speed continues to improve from the pre-school age up to early adulthood, as shown by a decrease in response time for a visual naming task during 4-23 years age. This improvement in naming efficiency was supported by alterations in coherence modulations among 13 parcel pairs, 3 of which showed significant age-related change (Figures 4–6). The most important pair consisted of left frontal operculum and left fusiform gyrus, because these were network hubs connecting to other brain regions, coherence modulation between them was significantly associated with mean response time, and significantly increased with age.

### Continuing Development of Naming Efficiency

Speech acquisition in children has been shown to occur at the level of whole-word production, rather than phonemic or segmental production (Redford, 2019). The fundamental unit of speech production in the context of language development is postulated to be a word, an ordered group of phonemes consistently representing an object or a concept, because the semantic associations cannot be encoded by isolated vocal features (Redford, 2019). From a developmental perspective, response latency is a biomarker of naming efficiency (Fried-Oken, 1984). In a study including 60 children, the mean response time for correct responses decreased from 1.4 to 0.9 s from 4 to 8 years age (Fried-Oken, 1984). However, this sample included 30 children with language impairment, whose response times (1.7 ± 1.6 s) were longer than typically developing children (1.3 ± 1.1 s, *p* < 0.001). In another study including 5-11 years-old school children with average intelligence, the response accuracy plateaued around 6 years age, but the naming latency continued to improve (Denckla and Rudel, 1974). Furthermore, naming speed was not related to the developmental order of acquisition of word categories, such as animals or tools. Our data is consistent with these studies, where mean response time decreased from 1.3 to 0.7 s in 4–23 years age range (model predicted values for age, Figure 2). Because we excluded the pictures that the patient was unable to name, our response times should be interpreted as being those for the correct responses. We also considered if the naming speed plateaus at a particular age, and *post hoc* fitted a non-linear (generalized additive) model to test this, but no improvement over linear regression was seen (details not shown). Therefore, our data supports continued improvement in naming speed between 4 and 23 years of age. We hypothesize that this improvement in naming speed has a role in the development of higher-order linguistic skills. This is supported by continued improvement in visual and auditory naming latency when accommodating for accuracy (Hamberger et al., 2018), change from concrete nouns in 5-9 year-olds to abstract nouns after 10–12 years age, with syntactic and grammatical use driven by personal and didactic exposures (Nippold, 2016), and age-related improvement in other measures of timed cognitive performance (Demetriou et al., 2002).

### Neuronal Circuits Supporting Naming Speed

We found that coherence modulations between certain brain regions were inversely related to response times (i.e., directly related to improvement in naming speed, Figure 4). These pairs mostly included connections between left peri-Rolandic cortex (frontal and central opercula) and left basal occipital-temporal region (fusiform and parahippocampal gyri, Figure 5). Also, coherence modulations between certain other parcel pairs were directly related to response times, implying that naming speed is related to desynchronization between these regions. These pairs mostly included parcels lying within canonical language areas (left frontal operculum, IFG pars triangularis, angular, and supramarginal gyri). Hence, it appears that naming speed is supported by synchronization of long-distance neuronal circuits between basal (left fusiform and para-hippocampal gyri) and frontal (left frontal operculum) language regions, while desynchronization of short-distance connectivity within the peri-sylvian language network. Specifically, the most important circuit supporting visual naming speed is that between left frontal operculum and left fusiform gyrus.

It is notable that we did not find any significant edges between left and right hemisphere parcels. This is probably because such analyses were limited to a small sample of patients having bilateral SEEG implants. Also, we used a strict correction for multiple comparisons, which limited the number of statistically significant parcel pairs. We think that the parcel pairs identified in this study as having significant association of coherence modulations with naming speed, and the resultant network graph (Figures 4, 5) probably represent the most important connections and circuit for development of visual naming, but not the only one. We have recently proposed a distributed model of spatiotemporal dynamics of visual naming involving 61 anatomic parcels (Ervin et al., 2021a). Hence, it may be assumed that significant connections exist between additional parcels, but may not contribute toward improvement in naming speed. It will be important to identify other anatomic regions and neuronal circuits, and how they relate to our findings, in a larger sample of patients in future studies. Convergence of multimodal data from lesion studies, fMRI, ESM, and HGM mapping shows that the common minimum region supporting visual naming is likely localized to left posterior temporal lobe (posterior superior and middle temporal gyri), adjacent left inferior parietal lobe (angular and supramarginal gyri), and left inferior peri-Rolandic cortex (IFG pars triangularis and pars opercularis, inferior precentral gyrus) (Skipper and Small, 2006; Hamberger, 2015; Arya et al., 2017). Furthermore, recent studies have shown that visual naming is a complex process with multiple temporal phases (Hamberger, 2015; Wang et al., 2016; Nakai et al., 2017, 2019; Forseth et al., 2018; Arya et al., 2019a). A study on propagation of HGM during visual naming showed that the occipital part of the left fusiform gyrus is typically activated immediately (< 10 ms) after image display while the temporal part within 200 ms during the visual decoding phase, but continues to show sustained HGM until after verbal response (Ervin et al., 2021a). Another study using multimodal mapping (fMRI, HGM, ESM) found that left fusiform gyrus, particularly the middle part, has a critical role in semantic access (Forseth et al., 2018). Whereas, the left frontal operculum has shown HGM typically between 800 and 900ms (after image display) in the pre-articulation phase just before the verbal response, and is known to be involved in phonological processing (Schwartz et al., 2012; Ervin et al., 2021a). Therefore, our data shows that strengthened direct connectivity between semantic and phonological nodes is the key driver of development of naming speed.

### Age-Related Dynamics of Neuronal Circuits

Coherence modulations between left frontal operculum and left fusiform gyrus, and between left frontal operculum and left posterior cingulate significantly increased with age (Figure 6). Increase in coherence modulations in both of these parcel pairs was associated with decrease in response time (improved naming speed). Therefore, it appears that age-related development of naming is supported by increased connectivity in a concurrent network among areas serving associative visual feature extraction and semantic access (fusiform gyrus), correlation of visual information with emotional and sensory inputs (posterior cingulate), and phonological processing (frontal operculum) (Chassagnon et al., 2008; Schwartz et al., 2012; Forseth et al., 2018; Palejwala et al., 2020; Ervin et al., 2021a).

A study using generalized q-imaging tractography and postmortem dissections showed that fusiform gyrus is the conduit for long-range association fibers from occipital cortex, with direct connections to frontal operculum *via* inferior frontal-occipital fasciculus (Palejwala et al., 2020). In future, it will be attractive to investigate if development of myelination in inferior frontal-occipital fasciculus correlates with age-related changes in coherence modulations between fusiform gyrus and frontal sites.

We also noted a significant age-related decline in coherence modulations between left angular and supramarginal gyri, which was associated with decrease in response time (improved naming speed). Given that these regions are involved in more sophisticated, multimodal, cognitive processing during the visual naming, we think that the efficiency of naming common environmental objects improves with age by elimination of intermediate steps which may be more related to contextual interpretation of the named object (Chassagnon et al., 2008; Binder and Desai, 2011).

### Limitations

Our study was done in DRE patients, raising usual concerns about heterogeneity of the sample (duration of epilepsy, location of seizure-onset zone, underlying pathology), and translatability to people without neurological disease. However, SEEG can only be performed in DRE patients being evaluated for epilepsy surgery. This may raise concern about the potential impact of epileptiform discharges on coherence modulations, however, our SEEG pre-processing excluded channels with frequent discharges. Another concern with SEEG is that of sparse sampling. While parcels were well-sampled (Figure 2), measuring coherence between pairs of parcels requires an individual patient to have electrode contacts in both parcels, which imposes a restriction. It is worth recalling that SEEG electrode placement is driven by clinical needs rather than experimental considerations. However, to allow informative analyses, we included only parcel pairs contributed by at least 5 patients. Historically, data from brain mapping in such patients has informed our understanding of brain function in neurologically healthy population (Lachaux et al., 2003; Hamberger, 2007). We performed this study using SEEG because of its superior spatiotemporal resolution compared to non-invasive modalities, and the ability to execute overt naming (Ball et al., 2009; Zaitsev et al., 2015; Ghuman and Martin, 2019).

A technical consideration that can impact SEEG coherence is the choice of montage, with every montage having certain limitations. For example, common average reference (CAR) may bias the coherence modulations toward cortices that are better sampled. While this may avoid phase effects, the power in CAR may be greater than many of the signals being analyzed (Fein et al., 1988). In a common scenario where adjacent electrode contacts may lie in different tissues (gray and white matter), the interpretation of bipolar montage becomes challenging. We decided to use referential montage, because the reference electrodes were distant from eloquent cortices. Thus, power in high-gamma frequencies which are important for cognitive processes, is likely to be greater in signals associated with cognition than in the reference. Also, our SEEG pre-processing were intended to help eliminate spurious connections due to contaminated reference. We used coherence as a measure of connectivity because it is perhaps the most widely used metric for quantifying synchrony between pairs of signals, and is easy to interpret (Bastos and Schoffelen, 2015). However, it is undirected, and susceptible to volume conduction effects (Bastos and Schoffelen, 2015).

Response latency for naming is also related to the frequency of a name in the language (for adults), stimulus context, and naming accuracy (Gardner, 1973; Denckla and Rudel, 1974; Rudel et al., 1980). By having the same starting set of images across all patients, eliminating pictures that the patient was unable to name, and recording under similar environment, we have tried to mitigate the effects of word usage, accuracy, and stimulus context. Response latency for picture naming is also influenced by literacy levels, and mutual information in HGM between parcel pairs is affected by IQ (Reis et al., 2001; Arya et al., 2019c). Therefore, we excluded patients with VCI < 70.

## Conclusion and Implications

We found that naming speed improves over 4-23 years of age, supported by network plasticity in the left cerebral hemisphere. Changes in coherence modulations between certain brain regions were associated with response times (latency) for visual naming, with the most important network hubs being left frontal operculum and left fusiform gyrus. Coherence modulations between left frontal operculum and left fusiform gyrus also showed a significant increase with age. Language development, insofar as it can be assessed by the ability to name pictures of familiar everyday objects, appears to occur by strengthening network synchrony among brain areas supporting visual feature extraction, semantic access, multisensory integration, and phonological processing, while simultaneous desynchronization of areas involved in higher-order cognitive processing.

In future, it will be desirable to extend this methodology of broadband coherence modulations to map neuronal circuits supporting other cognitive functions, and their age-related dynamics. It may be worthwhile to investigate if the time and/or frequency of maximal coherence modulation provides further neurophysiological insights, which will require a larger sample. Also, it will be important to explore the neurophysiologic role of anatomic areas which showed a significant association between response time and coherence modulations, but constituted stand-alone subnetworks, including those in the right hemisphere (Figure 5). We speculate that they may represent redundant areas for the development of visual naming circuits that may be recruited in patients with early childhood lesions in network hubs. Also, if this methodology can be used with non-invasive modalities, it can be harnessed to study development of brain networks in neurological disorders and healthy population.

## Data Availability Statement

The raw data supporting the conclusions of this article will be made available by the authors, for academic purposes, after the negotiation of appropriate agreements with the study institution.

## Ethics Statement

The studies involving human participants were reviewed and approved by Cincinnati Children’s Hospital Institutional Review Board. Written informed consent to participate in this study was provided by the participants, and/or their parents/legal guardians.

## Author Contributions

RA: study concept and design. RA, JB, CF, CS, HG, AB, JS, FM, JL, and KH: data acquisition. RA, BE, AB, and LR: data analysis. RA and PH: statistical analysis. RA, BE, HG, AB, and KH: data interpretation. RA, BE, JB, HG, AB, LR, JS, PH, CF, CS, JL, FM, TG, and KH: drafting and revision of the manuscript for content. All authors contributed to the article and approved the submitted version.

## Conflict of Interest

All commercial software used in this research were covered under appropriate end-user licenses. Python 3.x (Anaconda distribution) and R (version 4.x) are open-source programming languages. The methodology of non-parametric clustering of high-gamma activations and broadband coherence modulations in intracranial EEG is covered by patent US62/982,148 (pending). RA receives research support from NIH NINDS R01 NS115929, Cincinnati Children’s Research Foundation (Research Innovation Project Grant), University of Cincinnati Center for Clinical & Translational Science & Training (Pilot Collaborative Studies Grant) and has received support from Procter Foundation (Procter Scholar Award). The remaining authors declare that the research was conducted in the absence of any commercial or financial relationships that could be construed as a potential conflict of interest.

## Publisher’s Note

All claims expressed in this article are solely those of the authors and do not necessarily represent those of their affiliated organizations, or those of the publisher, the editors and the reviewers. Any product that may be evaluated in this article, or claim that may be made by its manufacturer, is not guaranteed or endorsed by the publisher.
