# Supplementary material for: Neuronal Circuits Supporting Development of Visual Naming Revealed by Intracranial Coherence Modulations
Source: Front Neurosci. 2022 May 19;16:867021. doi: 10.3389/fnins.2022.867021 (PMC9160526; doi:10.3389/fnins.2022.867021)
Supplement: Supplementary file 1 [file Table_1.DOCX]

Supplementary Table 1

Demographic and clinical details of individual patients

| Patient | Age (years) | Sex | Hand | fMRI | Seizure Onset | Electrodes (n) | Electrode Contacts (n) | SEEG Lobar Coverage | Seizure onset zone |
| --- | --- | --- | --- | --- | --- | --- | --- | --- | --- |
| 1 | 16.1 | m | R | L | 10y | 11 | 124 | LF, LP, LT | L post-central gyrus/peri-rolandic cortex |
| 2 | 14.9 | m | R | NA | 5y | 12 | 94 | LT, LP, LF | L hippocampus |
| 3 | 21.2 | f | R | L | 13y | 10 | 102 | LF, LP | L frontal |
| 4 | 16.9 | f | R | NA | 3y | 16 | 182 | LF, RF, LT, RT, LP, RP | Unknown |
| 5 | 7.1 | m | R | NA | 5y | 12 | 127 | LF, LT, LP | L inferior precentral gyrus |
| 6 | 10.6 | m | R | L | 9y | 11 | 136 | RTPOJ, RT | R hippocampus |
| 7 | 12.2 | f | R | L | 8y | 11 | 118 | LT/LTOJ, LP, LF | L anterior temporal |
| 8 | 14.9 | m | R | NA | 9y | 14 | 126 | RO/RTOJ, LO/LTOJ, RP, LP | Independent L/R occipital |
| 9 | 7.7 | m | R | NA | 3.5y | 15 | 199 | LO, RO, LT, RT, LP, RP | L occipital |
| 10 | 20.8 | f | L | L | 15y | 9 | 123 | LP, LO, LT | L inferior parietal lobule, SMG |
| 11 | 10.7 | m | L | NA | Neonatal | 16 | 186 | LO, LP, LT, RO | Independent L/R occipital |
| 12 | 16.4 | f | R | L | 7y | 9 | 94 | RF, RT | R anteromesial temporal |
| 13 | 4.9 | m | L | L | Neonatal | 14 | 154 | LF, LP, LT | Independent L frontal operculum, pre-central gyrus, STG |
| 14 | 11.9 | m | R | NA | 7y | 18 | 208 | LF, RF, LT, RT, LP, RP | Unknown |
| 15 | 12.6 | m | R | NA | Neonatal | 10 | 87 | RO, RT, RP | R inferior parietal lobule |
| 16 | 16.5 | m | B | L | 4y | 10 | 108 | LT, LF, LP | Left parietal and insular cortices |
| 17 | 18.9 | m | A | NA | 6y | 20 | 253 | LO, RO, LTOJ, RTOJ, LP, RP | L occipital |
| 18 | 7.8 | m | R | L | 4y | 11 | 112 | LT, LP, LF | L hippocampus |
| 19 | 16.8 | m | R | NA | 11y | 13 | 120 | RF, RT, RP | R anterior insula |
| 20 | 11.6 | m | R | L | 7y | 13 | 140 | LT, LF, LP | L posterior STG and MTG |
| 21 | 17.1 | m | R | L | 13mo | 12 | 152 | LT, LF | L posterior inferior temporal gyrus |
| 22 | 16.5 | m | R | L | 8y | 9 | 97 | RT, RF | R posterior STG |
| 23 | 15.2 | f | R | L | 4y | 13 | 152 | RP, RT | R inferior post-central gyrus |
| 24 | 19.0 | f | B | L | 14y | 11 | 112 | LT, LTOJ, LF, LP | L hippocampus |
| 25 | 11.7 | m | R | L | 8y | 12 | 146 | LF, LT, LP | L hippocampus |
| 26 | 3.9 | m | R | NA | 2y | 13 | 132 | RF, RP, RT | R parietal |
| 27 | 10.9 | m | R | NA | 6mo | 8 | 84 | RF, RP, RO | R posterior cingulate |
| 28 | 11.0 | f | R | L | 7y | 10 | 116 | LT/LTOJ, LF, LP | L posterior temporal, temporo-parietal junction |
| 29 | 17.0 | m | L | L | 2y | 4 | 42 | LP | L inferior postcentral gyrus |
| 30 | 10.8 | m | R | L | 6y | 8 | 90 | LF, LP, LT | L middle cingulate |
| 31 | 23.1 | m | R | NA | 15y | 12 | 166 | LF, RF, LP, RP | L superior frontal gyrus |
| 32 | 13.6 | f | R | L | 1y | 9 | 100 | LT, LF, LP | L hippocampus |
| 33 | 18.3 | f | R | L | 6y | 14 | 116 | LF, LT, LP | Multifocal |
| 34 | 19.6 | m | R | L | Neonatal | 15 | 150 | RF, RP, RT | R peri-rolandic |
| 35 | 6.6 | m | R | NA | 3y | 12 | 152 | RP, RT, RF | R hippocampus |
| 36 | 14.9 | f | R | NA | 10y | 11 | 117 | LF, RF, LT | L inferior frontal gyrus |
| 37 | 19.6 | f | R | NA | 9y | 19 | 194 | LF, RF, LT, RT, LP | L mesial temporal lobe |
| 38 | 14.9 | f | R | L | 10y | 12 | 137 | RT, RP, RF | R posterior insula |
| 39 | 4.8 | f | R | NA | Neonatal | 13 | 113 | RT, RP, RF | Unknown |
| 40 | 10.5 | m | R | L | 5y | 13 | 154 | LF, RF, LP, RP, LT, RT | R parietal operculum |

[Abbreviations: f female, F frontal, fMRI language lateralization with functional magnetic resonance imaging, L left, m male, mo months, MTG middle temporal gyrus, O occipital, P parietal, R right, SEEG stereo-electroencephalography, SMG supramarginal gyrus, STG superior temporal gyrus, T temporal, y years]
